# Paradigms unfolded – developing, validating, and evaluating the Medical Education e-Professionalism framework from a philosophical perspective

**DOI:** 10.3389/fmed.2023.1230620

**Published:** 2023-10-19

**Authors:** Shaista Salman Guraya, Denis W. Harkin, Muhamad Saiful Bahri Yusoff, Salman Yousuf Guraya

**Affiliations:** ^1^Royal College of Surgeons Ireland, Medical University of Bahrain, Bahrain, Bahrain; ^2^Department of Medical Education, School of Medicine, University Sains Malaysia, Kelantan, Malaysia; ^3^Faculty of Medicine and Health Sciences, Royal College of Surgeons Ireland, Dublin, Ireland; ^4^College of Medicine, University of Sharjah, Sharjah, United Arab Emirates

**Keywords:** paradigms, pragmatism, mixed-methods research, Medical Education e-Professionalism framework, e-professionalism

## Abstract

In order to ensure a strong research design, literature stresses the adoption of a research paradigm that is consistent with the researcher’s beliefs about the nature of reality. In this article we provide an overview of research paradigm choices in relation to the creation of a Medical Education e-Professionalism (MEeP) framework discussing the research design, research methods, data collection and analysis to enhance the transparency of our previously published research. The MEeP framework was conceived to help Health Care Professionals (HCPs) safeguard the construct of professionalism in the digital context. This entire process was heavily informed by wider readings and deliberations of published literature on e-professionalism. Although the MEeP framework research journey has been published, the paradigms approach was not discussed in any detail. Considering that one of the duties of medical educator is to balance the service and science by bringing the theoretical underpinnings of one’s research to public attention and scrutiny so as to nullify the notion of ‘weak’ research. We were compelled to unfold this paradigm story of the MEeP framework in a detailed manner. In an effort to make our research both robust and effective, this study portrays a philosophical approach to guide future research designs and methodological choices by detailing our rationale for pragmatism as a choice of paradigm.

## Introduction

In educational research, Mackenzie and Knipe (1) and Morgan (2) used the term paradigm to express the researcher’s ‘*worldview*’. Whereas a more elaborative definition of paradigm was introduced by Morgan (2) as a “*set of assumptions, research strategies and criteria for rigor that are shared by the research community*” and “*a system of ideas, or world view used by researchers to generate knowledge*.” Paradigm represents the ‘*ultimates*’ encompassing ontological, epistemological, and methodological beliefs of a researcher, which often indicates the researcher’s viewpoint (3). This highlights the importance of paradigm which yield beliefs and directs the crucial steps in planning and execution of the research process. This step also establishes the researcher’s philosophical orientation which has significant bearing on the research process and design (4). In order to ensure a strong research design, published literature stresses the adoption of a research paradigm that is consistent with the researcher’s beliefs about the nature of reality (5). To accomplish this goal, Levers (6) explained that selection of paradigm aligns with researcher’s perspectives of reality and should be made explicit in the process. Lincoln and Guba (7) communicated that a paradigm comprises of four elements, namely, epistemology (our ideas about knowledge), ontology (our existence), methodology (research design and methods) and axiology (the role of values in inquiry) (8, 9). As suggested in the literature, it was paramount to have a close-grained grasp of these elements to identify our position on a particular research paradigm, the underlying guiding assumptions, beliefs, norms, and the values of the chosen paradigm (10). This would help establish an understanding of our research while narrating the journey of the development of the MEep framework (11).

As our research centred on the ever-evolving field of the digital world and its impact on professionalism of healthcare professionals, it manifested new and evolving professional dilemmas and potential erosion of professionalism perspectives. There was no obvious philosophical paradigm to align with our research due to nature of data (enumeration and explanation) required. In order to conduct a cohesive and valid research study, we explored methodological paradigms along with ontological and epistemological perspectives by blending different knowledge claims and inquiry strategies. Similarly, having to select an appropriate lens for this research placed us at the crossroads of these research paradigms where a sharp division became unnecessary. Both epistemological and ontological aspects were needed for the evaluation of e-professionalism concept and later for the development and understanding of the essential attributes required for an effective digital professional. We adopted a complex multi-stage research design and used data from a systematic review, self-reported survey, and Delphi technique and experts’ reviews to develop, validate and evaluate the MeEP framework. The results of all stages of the MeEP framework journey have been published recently (12–15). The MEeP framework’s journey sheds light on professional competencies in terms of characters, characteristics and identifies constructs which can be inculcated in future healthcare professionals (HCPs) to become digitally skilled. However, it became evident that a pluralistic framework for HCPs to navigate through the dilemma of thriving in the digital realm was needed. Especially since one of the duties of a medical educator is to balance the service and science by appreciating the theoretical underpinnings behind one’s research to public so as to nullify the notion of ‘weak’ research (10, 16). Therefore, in this research we aim to provide an account of the four elements of the paradigm followed by a structured process of rationalizing the paradigm choice in the journey of the MEeP framework.

### Epistemology

Schwandt (17) defined epistemology as the study of the nature of knowledge and justification in line with Crotty’s approach (18) “*a way of understanding and explaining how I know what I know*.” Whereas Denzin and Lincoln (3) described it as “*a relationship between the knower and the knowledge*” investigating how the researcher makes meaningful sense of the world. This understanding can oscillate between positivist – “*knowledge as personal, subjective and unique*” to interpretivist – “*knowledge as hard, objective and tangible*” (19). Positivists dwelling on the philosophical perspectives base their knowledge on the external existence of reality. By employing quantitative methods (surveys and experiments), they infer using replicable statistical analysis thus dissociating the researcher from the whole process. For positivists, the formulation of hypotheses is crucial for the verification of knowledge. While on the other end of the spectrum, Interpretivists, or social constructivists, view knowledge as complex context-specific socially constructed entity (20). Interpretivists acknowledge the significance of history and practical experience in the advancement of knowledge. They believe in the crucial role of research participant and how one’s expertise and cognitive base influence the whole research process. They strongly believe in the role of researcher’s cognitive maturity that shapes the understandings and discussions with the study participants in each context. On the other hand, realism, which shares similarities with positivism, adopts a scientific approach to developing knowledge. However, realists being on the anti-positivist spectrum rely on triangulation to unveil the truth. Realists highlights the role of interpretations in the context of social environment (21). In articulating answers to the above questions, there are further terminologies coined to define subcategories of knowledge, *intuitive knowledge* (knowledge of beliefs, faith, and intuition), *authoritative knowledge* (knowledge gathered from people, books, leaders in organizations), *logical knowledge* (knowledge emphasizing reasoning), and *empirical knowledge* (knowledge of experiences, and demonstrable objective facts) (22). This, type of detailed discussion is not within the scope of this article. However during the MEeP journey we used the concept of epistemology lauded by Saunders et al. (23) as “*the acceptable knowledge in the field of study*,” a useful classification of objectivism, constructionism and subjectivism (18).

### Ontology

Ontology is regarded as “*the study of being*” as described by Crotty (18) and “*raises basic questions about the nature of reality and the nature of the human being in the world*” (3). It focuses on understanding the nature of reality and the assumptions we make about it. In other words, ontology examines the objective or subjective aspects of social entities and unfolds the true dynamics of the things. The ongoing ontological debate is whether social reality is individually constructed from consciousness or is it external and imposed on consciousness. In other words, do things exist independently of our mind, or is our world something constructed from our thoughts (19)? This implies that there are two ontological perspectives: realism and idealism. Realists argue the organic nature unrestrained of human discernment. On the other hand, idealists holding the opposing view, confess and endorses the allegory of Plato where human mind constructs its own reality using preconceived notions of shadows. Philosophical assumptions about the nature of reality play a pivotal role in our understanding and on the inferences drawn from the data. These orientate our thoughts about the research problem, its significance and our approach to problem solving. Hence, ontology plays an essential role in our understanding of the things that constitute the world (24). In MEeP framework journey the ontological position is clear by using external multiple views to best answer the research question.

### Methodology

‘Methodology’ refers to “*the process, principles and procedures by which a researcher approaches problems and seeks answers*” (25). While Langdridge (26) refers to methodology as a term rather than a process as a “*general way to research a topic,” while “method is the specific technique (s) being employed.*” Hence, broadly speaking methodology is about research design and methods describe the approaches, and procedures. This includes data collection, participant sampling techniques, instruments used and data analysis conducted so as to answer the research question ensuring a substantial contribution to knowledge. In summary, methodology narrates the systematic process used in conducting the research including assumptions made, limitations encountered and addressed. Both methodology and methods used in this research are discussed in the latter half of this article.

### Axiology

Axiology is defined as “*the philosophical approach to making decisions of value or the right decisions*” (27). Axiology involves the examination of values and the foundation upon which a researcher makes value judgments. A researcher’s personal values, beliefs, and experiences can influence their research and may impact their ability to remain unbiased when it comes to the concept of value. There are two axiological positions: positivism, which emphasizes value-neutrality, and interpretivism, which acknowledges the presence of values in research. To achieve this understanding of definition and evaluation of the concept of right and wrong pertinent to the research is essential. Simply stated, it’s about the ethical behaviour maintained during participant recruitment, data collection and the dissemination of findings to the wider audience. This understanding of axiology dates back to Mill’s utilitarian ethics with an understanding that all humans have dignity and the right to choose, which should be respected (28). Keeping this in mind, four principles namely; privacy, accuracy, property and accessibility were upheld while dealing with research participants and data (29).

After identifying the four basic elements which form a paradigm, the next step was to designate an epistemology, ontology, and axiology to assist in developing the methodology (2). By seeking insights from Tashakkori et al. (30) whose advancing work on taxonomies of paradigms from pre-existing research by Candy (31) added a new pragmatic paradigm to the original taxonomy set. They borrowed elements from the Positivist, Interpretivist, and Critical paradigms (31). They kept “the research problem” as the central pivot, which we used while focusing on shaping the attitudes, values, beliefs, thoughts, and behaviour of medical students and changes on various levels, while reconciling eclectic views on how e-professionalism is understood, discovered, learned, valued, justified, and verified, thus challenging concrete ideas of science (32). For the purpose of this research, we broadly related our approach to ‘pragmatism’ for interrogating and evaluating ideas and beliefs regarding e-professionalism and their practical utility in maintaining the societal contract of profession. However, before we rationalize the principles of pragmatism in the context of this research, lets describe the evolution of this paradigm.

## Pragmatism – a philosophical paradigm

Dewey (33) conceptualized epistemology as the “*theory of inquiry*” comprising of experiencing, knowing, and acting which demands a dynamic view of social life. Obviously, unravelling of ‘*truth*’ about this dynamic world cannot be accessed by virtue of single scientific method. Historically, science is an amalgam of various collective truths ranging between objective and subjective assumptions about *ontology* (our existence), *epistemology* (our ideas about knowledge), *research methods*, and *human nature* which forms the basis to challenging the solid foundations of science (8, 9). Many philosophers agree that relational epistemology, non-singular reality ontological viewpoint, a mixed-methods methodology and value-laden axiology were the best way forward in understanding human behaviour (34–36). Drawing on the works of Creswell, Tashakkori, and Teddlie (36, 37) we identified the following characteristics of pragmatic research for MEeP framework journey;

- Rejection of the positivist notion (facts and measurable entities).
- Rejection of absolute post-positivist and constructivist notions (reality and cognition).
- An emphasis on workability in research.
- Choice of research design and methodologies that work for the research question/s.
- Use of the most feasible methodological approaches for knowledge acquisition.
- Choice of research methods well aligned with the purpose of research.
- Triangulation; identifying useful point of connections within the research and to avoid potential biases to enhance the quality of research.

It became obvious that pragmatism was an inclusive approach, which simultaneously appreciated the existence of reality (objectivism) and the individualized worldview (subjectivism) bringing multiple explanations and interpretations of science (23). Table 1 summarizes the nature of pragmatism on the above mentioned four elements of epistemology, ontology, methodology and axiology adopted during the MEeP framework journey.

**Table 1:** Summary of four elements of pragmatism for developing the MEeP framework.

Elements	Pragmatism
Epistemology	Depending upon the research question, focusing on the practicality and applicability of the research objectives and subjective and objective approaches can provide admissible knowledge integrating different perspectives in data interpretation
Ontology	Integration of multiple external views chosen in answering the research question
Methodology	Mixed or multiple method designs, quantitative and qualitative approaches
Axiology	Role of values in results interpretation, while the researcher adopts both objective and subjective viewpoints

Appreciating that pragmatism was not aligned to any one system of philosophy gave us the freedom of choice for methods, by viewing the social reality from a different lens that yielded transferable context rich findings (38). Additionally, the pragmatism ‘*world-view*’ dissipated the clear divide between methodological choices, logic and epistemology sufficiently to pacify the paradigm wars (33, 39, 40). In an attempt to safeguard this important societal contract and understanding the phenomenon of e-professionalism viewing this problem through the philosophical lens of ‘*pragmatism*’ seemed most appropriate.

MEeP framework came into being while keeping in mind how a model can be conceptualized, developed and applied to change professional behaviour of health care professionals about e-professionalism. To achieve this, following questions were probed in a phased manner.

1. What is the nature, degree, and professional use of social media by the undergraduate (UG) medical students?
2. What are the definitions of the constructs for the new proposed MEeP framework?
3. How can the key elements of new MEeP framework be identified?
4. Does the new MEeP framework have sufficient content and response process validity?
5. How does the MEeP framework impact on the reaction, learning and behaviour of learners?

This article provides our philosophical paradigm overview of the materials, methods, and data analysis approaches used while developing, validating, and evaluating Medical Education e-Professionalism (MEeP) framework in a phased manner (Table 2).

**Table 2:** Summary of the phases involved in the research study.

	Phase I – Development		Phase II – Validation	Phase III – Evaluation
	Study 1	Study 2	Study 3	Study 4
Design	Convergent parallel		Concurrent embedded	
Rationale	An exploratory step in the development of the MEeP framework	A framework for healthcare professionals to help cope with the challenges of medical professionalism in the digital realm	To seek reassurance by consulting experts regarding the validity of the MEeP framework	To measure the impact of MEeP framework in changing professional behaviours of learners in the digital world
Aim	To identify the key concepts and threats to professional identity in the era of e-professionalism	To develop the MEeP framework	To validate the MEeP framework	To evaluate the MEeP framework at the behaviour level of Kirkpatrick’s pyramid using Theory of Planned Behaviour
Approach	QUAL	QUAL-QUAN	QUAN-QUAL	QUAL-QUAN
Sampling strategy and participants	*SPIDER (*n* = 44 studies)	Convenience (SNSME *n* = 381) Purposive (Delphi *n* = 15)	Purposive (*n* = 6)	Convenience (*n* = 59)
Data collection	PRISMA technique	Online questionnaire and online multi-round iterative approach for Delphi	Online meeting with experts and online survey	(Online) Pre-post workshop survey and breakout room discussions
Analysis	Thematic	Descriptive and thematic analysis using grounded theory approach	Descriptive and content analysis	Descriptive and structural Equation Modelling – Thematic analysis

*PRISMA, preferred reporting items for systematic reviews and meta-analyses. *SPIDER – S, sample; PI, phenomenon of interest; D, design; E, evaluation; R, research type.

## Applying the principles of pragmatism in context of MEeP framework

### Research design

Keeping our pragmatist view of the world in mind, a unique literature review in the form of concept analysis (In press) prompted us towards developing a well-framed research objective, a concise research question and a well-aligned methodology. A significant and thorough literature review accomplished by assessing the theoretical published literature enabled us to refine the research objectives. Using the ‘*what works*’ Pragmatic approach helped us to unpack the ‘truth’ of the emerging social reality of e-professionalism and avoiding the ‘either-or’ qualitative-quantitative polemic through the pragmatist and a pluralist research philosophy developed by Bilau (41). When making the ‘theory choice’ decision, a number of concerns in relation to prior conceptualizations of three of the fundamental elements: *ontology*, the perception of being subjective or objective in the real world; *epistemology*, the realm of understanding from reflections; and *axiology*, the researchers’ persona of opinions and beliefs became evident (19).

Pragmatism’s inherent focus was on the experience and action of the research question thus prompting us to look for multiple perspectives in developing MEeP framework (12, 13). Thus, revealing distinctions between how different stakeholders (medical staff and students) and published literature enact evaluations regarding e-professionalism. Likewise, the processing of data collection and results analysis revealed the peculiar differences and diversity of views between the different stake holders. Respecting the diversity of understandings, validation of the MEeP framework by panel of experts’ relevant and relatable facets of our research were highlighted and endorsed (14). Building in scope to evaluate the MEep framework by introducing it to those whose professional behaviour had not been consolidated. Using Kirkpatrick’s model with a sound theoretical underpinning of Theory of Planned Behaviour (TPB) we measured behavioural changes of digital natives (15). Pragmatism framed the appropriate methodology by unpacking the different aspects of this phased research questions at the design stage (Table 3).

**Table 3:** Relationship of pragmatic paradigm elements to phased research questions.

Research questions	Ontology	Epistemology	Axiology	Study
Phase I – Framework development – To develop a Medical Education e-Professionalism (MEeP) framework which can describe healthcare professionals’ expected conduct using SNSs. To develop a framework for healthcare professionals coping with the challenges of medical professionalism in the digital realm.				
What is there in the published literature related to suggested e-professionalism?	Knowledge – existing social phenomena. Idealism was applied	Qualitative literature review – Interpretative approach	Value – laden	Study 1
What is the degree, nature (social or educational) and professional use of SNS?	Knowledge – existing social phenomena. Idealism was applied	Reality – a result of the human mind, data from stakeholders’ opinion – interpretivist approach	Value – laden	Study 2
What are the desired values and behaviours of digital professionalism that are needed for maintaining digital professional identity?	Knowledge – outside the social phenomena. Realism applied	Experts’ opinions – Pragmatist’s approach	Value – free	
Phase II – Framework validation – To perform Content Validity Indexes (CVIs), Face Validity Index (FVI), and inter-rater reliability of the MEeP framework				
Does the MEeP framework has sufficient content, face and response process validity?	Knowledge – outside and inside the social phenomena. Realism applied	Experts’ opinions: rating scales and free text comments – Postpositivist approach dominated	Value – free but the content analysis f free text comments value laden	Study 3
Phase III – Framework evaluation – To determine educational impact of the MEeP framework among medical students				
How does the MEeP framework impact the reaction, learning and behaviour of learners?	Knowledge – outside and inside the social phenomena. Realism applied.	Reality – a result of the human mind, data from stakeholders’ opinion using TPB – Interpretivist approach. But digital natives’ perceptions and opinions – Pragmatist’s approach dominated	Value – laden with surveys and deductive-inductive thematic analysis	Study 4

### Sampling strategy

In our sampling strategy, pragmatism was the key instrument in the selection of the most suitable participants. As narrated earlier, this research aimed to explore the phenomenon of e-professionalism and its impact on the societal contract. Pragmatism guided us into unravelling this abstract concept of e-professionalism as well aiding the development and evaluation of the new MEeP framework. Revisiting the key principle of pragmatism; inquiry as an experiential process we placed an emphasis on actionable research knowledge, by ensuring sampling decisions would be unbiased and adhered to the pragmatism dogma. Our objective was to explore what different attributes were necessary for an individual to be digitally professional and to develop a framework containing those key attributes for other healthcare professionals. Keeping this in mind and using a convenience sampling strategy (42), the use of Social Networking Sites (SNSs) by undergraduate medical students was probed (13). Convenience or opportunity sampling is often used by researchers as it aids in the selection of a defined population in this case undergraduate medical students (43). All registered undergraduate medical students at the Royal College of Surgeons Ireland Bahrain were approached by gatekeeper which also highlighted our axiological stance on this paradigm.

However, the sampling strategy was changed during the Delphi study (13) and expert validation (14). Pragmatism helped us to develop a more targeted selection of participants to allow for the exposure of a range of perspectives by searching for information rich respondents. Purposive or judgment sampling; is a technique known for the deliberate freedom of choice in selecting participants with peculiar characteristics (44). This sampling technique offers a targeted selection of participants aimed to establish macro–micro linkages by juxtaposing a diversity of perspectives. Patton (45) described these individuals as having a ‘*personal factor*’ called as “*a caring trait about the evaluation and findings it generates*.” These individuals included academics, clinicians, executives (deans and vice deans) and professionalism subject experts spanning from various generational archetypes. The evaluation of MEeP framework however utilized a convenience sampling strategy (15). Adhering to pragmatism principles aided the mapping, triangulation and sequencing of different steps used to answer our research question.

### Data collection

In the choice of methods of data collection, the pragmatic approach gave us the power to exercise researchers’ subjectivity during the observation process with a very small role for pre-defined theoretical classifications of resultant outcome interpretations, a possible limitation of our research. Focusing on the purpose of research; considering social, historical, and different constructs of professionalism, we integrated multiple realities and verified the assumptions by numerical calculations to create meaningful concepts (46). As Creswell (47) rightly argued pragmatism tackles problem-centred, pluralistic, real-world practice-orientated phenomenon which highlight the consequences of actions. Using the four key elements of the ‘pragmatism’ continuum (Table 1) developed by Creswell (38), we organized the research methods using both quantitative and qualitative approaches with inductive and deductive considerations using epistemological relativism (38).

### Mixed-methods approach

We adopted a mixed-methods approach based on our epistemological relativism and the complexity of e-professionalism. The mixed-methods design belongs to a specific set of methods combining enumeration and description and thus creating a synergistic model of understanding and knowledge creation (38, 48). The value of mixed-methods studies has been progressively recognized within the field of medical education as a means to facilitate researchers who want to examine both breadth and depth of a specific issue or phenomenon (48). Using the Johnson et al. (49) definition of mixed-methods research, we integrated ‘*theory and practice*’ by blending numerous frame of references, stances, perspectives, standpoints and views using the optics of qualitative and quantitative research, which complemented both our epistemological position and justified the rationale behind MEeP framework journey (50). The choice of mixed-methods approach was warranted for several reasons, two of which had resonated with us. First, the results from our first study (12) were used to inform and guide the method for the subsequent study. Although there is a plethora of research on the constructs and framework development of medical professionalism, very few studies have reported on e-professionalism and constructs needed to professionally navigate the digital world (12). A great number of the studies probed the opinions and perspectives of the participants regarding professionalism in the digital world. Some studies investigated desired online activities, professional online presence, and an understanding into the guidelines on professional use of digital media. Numerous examples from the literature indicated the erosion of professional integrity while in the digital world and have signalled blurred boundaries between professional and unprofessional lives (51–55). Despite the extensive epistemological description, the existing body of literature lacked both the realistic and idealistic ontological perspectives. In this context, our two studies made a unique contribution to the field of e-professionalism (12, 13). Second, based on the notion of complementarity, whereby a method was chosen to enhance, expand or clarify existing results using a different strategy (56), we used both quantitative and qualitative approaches synchronously to identify and highlight the utility of the MEeP framework and verify these findings from numerical and subjective positions.

Quantitative research does not tend to follow ‘traditions’ explicitly as clinical researchers consider case series, cross-sectional and case-control and randomized controlled designs as quantitative, while social scientists consider experiments and surveys as quantitative research (47, 57). Relying heavily on reductionism, quantitative methods categorize human dilemmas and experiences into numerical values. While qualitative approach using the psychodynamic lens views human dilemmas as too complex to reduce into numbers or categories (58). This approach explores the uncertainty especially of ‘immature’ concepts, complex human intentions and motivations using ‘case-oriented’ research (59–61). Creswell (47, 62) outlined five main qualitative traditions of narrative, phenomenology, grounded theory, ethnography, and case studies. To add further confusion, in the field of health research, another set of qualitative subdivisions have been made using the terms field-, action-, or library-based approaches. This dichotomy has led to an unhelpful polarizing of epistemologies between those considered positivists (biomedical orientation) on one side and those considered interpretivism (humanist orientation) on the other. This zealous divide between quantitative and qualitative approaches described by Bergsjø as a ‘*phony war*’ dates back to the 1800s when extreme polarisation of positivism vs. interpretivism paradigms ensued (63). In our research, we explored both literature and ground realities around the topic of e-professionalism, erosion of professionalism in the digital world and the reasons why digital natives are not successful in safeguarding this vital construct.

During this exercise, we combined quantitative and qualitative research techniques and methodological approaches and merging concepts into a single research strategy with an idea of complementarity, timing, point of integration, typological and interactive perspectives (64, 65). In a research program, mixing of methods can span across studies however the strategy of mixing should be explained with firm justifications regarding sequential order. Qualitative and quantitative studies can be undertaken concurrently with the qualitative first, or quantitative first, or convergently when the qualitative and quantitative parts are conducted at the same phase of the research study (66, 67). Priority (equal, or either method prioritised), and the rationale regarding nature and timing of integration (full or partial, during data collection, analysis, or interpretation) (61, 68, 69). Keeping in view the different paradigmatic origins of qualitative and quantitative methods, caution must be used when conducting mixed-methods by avoiding a sharp dichotomy between their values and methods.

**Figure 1:**
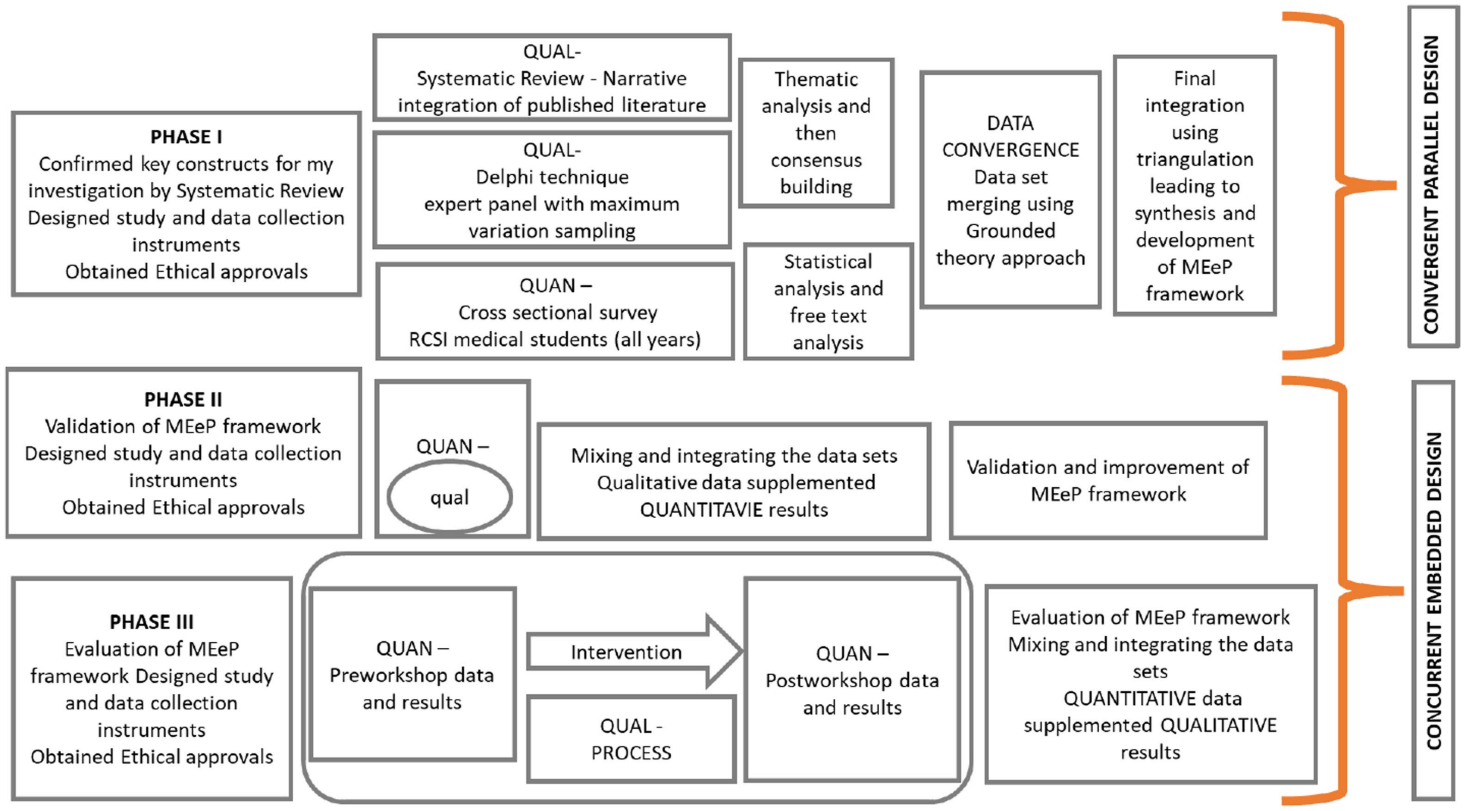
A visual illustration of the mixed-methods approach used in this phased research.

Developing the MEeP framework with a mixed-methods design involved the use of both qualitative and quantitative methods to explore the phenomena of e-professionalism in the context of digital natives’ degree, extent, and nature of the use of Social Networking Sites and experts’ opinions about the desired attributes in value, behaviour and identity constructs (13). This mixed-methods approach had similarities with the methodology used by some early research on conventional professionalism (70–74). However, a seminal work on e-professionalism by Ellaway (75) relied heavily on the review of existing literature. The use of both methods in our study design enabled us to generate new inductive knowledge, quantify and describe the phenomena of interest and generate new insights and hypotheses. This was not a linear process, rather findings from each study typically influenced and informed the idea and design of more than one subsequent studies. Furthermore, the point of integration for different studies varied as described by other researchers. In order to achieve a successful integration of tangible relationships at various levels of methodology, the data analysis and interpretation and research rigor were maintained (68, 76, 77). By using Tashakkori’s (38) approach, data was collected, analysed, results integrated, and conclusions were made using both quantitative and qualitative approaches in all three phases of our research (Figure 1).

As shown in Table 4, the pragmatic approach was contextual and application oriented. These reflections on the current challenges in medical education through a pragmatism lens provided us with an inclusive methodology leading to the production of a comprehensive and elaborative set of solutions to the research question.

**Table 4:** Components of pragmatic research and their key features using illustrative examples from the MEeP framework journey.

Pragmatic component	Key features	Purpose	Illustrative example from the MEeP journey
Approach	Focus on application, context and usefulness	To address specific questions and practical needs	To understand attitudes, values, beliefs, modes of thought, and behaviour of medical students in digital context (12, 13)
Models and frameworks	Focus on key issues for success, important to policy makers and stakeholders	Without overly complex theoretical underpinnings keeping contextual relationship in mind	The ultimate impact on various levels, while reconciling eclectic views on how e-professionalism is understood, discovered, learned, valued, justified, and verified (13)
Designs	Focus on resources, context, replication and applicability of results	Address issues prevalent in multiple heterogeneous settings in real world with a rigor and relevance having few exclusion criteria	Development (e-Delphi) and validation (experts in an online manner) and evaluation (13, 14)
Measures	Reliable, valid, feasible, relevant, actionable, brief, broadly applicable, user-friendly and sensitive to change	Feasible and actionable in real world settings	Detailed and reproducible methodology, validated questionnaires, inductive-deductive-abductive approach to results interpretation (13–15)

### Reflexivity

Reflexivity is defined as a continuous reflective process by the researcher to critically analyse attitudes, values beliefs and behaviours that can affect the interpretation of the study outcomes (78). Mixed-methods research goes beyond merely mixing quantitative and qualitative approaches while collecting and analysing data to maintain rigor and relevance, it also demands the researcher’s reflexivity. Throughout our research, pragmatism nudged the researchers to adopt a reflexive stance during all stages of data collection. The role of reflexivity is essential in the research process to create a nuanced and context-specific understanding of the e-professionalism concept (79). During the systemic review (12) the need to develop a link between published literature, quantitative (SNSME) and qualitative (Delphi) (13) became clear in bringing new dimensions to the MEeP framework constructs. During the Delphi, reflexivity was undertaken through the circulation of plain language questions and later the interpretation of text generated codes, descriptors, and themes. These were fed back to the respondents to refine and endorse in an iterative manner. During the validation of the MEeP framework (14) a real-time online validation process using both rating scales and free text comments was another way to improve reflexivity. Pragmatist inquiry embedding the ethical considerations enabled us to adopt, adapt and involve respondents with different levels of knowledge and experience. The evaluation of the MEeP framework (15) was carried out by adhering to the ‘*moral responsibility*’ principle of pragmatism, we presented the knowledge which has a promising future application in the field of medical education. Keeping the integral element of researcher’s subjectivity as part of pragmatism, ambiguities were avoided in data analysis by evaluating the MEeP framework against Kirkpatrick’s model using Theory of Planned Behaviour (TPB) (80).

### Analysis, dissemination, and conclusions

In our work, pragmatism was a guiding force influencing the approaches and techniques while analysing data and drawing conclusions. Using the ‘meta-interface’ approach where purposeful consideration was given to the evidence obtained about the phenomenon of interest using qualitative and quantitative types of data (46). This unique approach identified contradictory and confirmatory elements of evidence and led to a revised understanding of e-professionalism. While developing the MEeP framework (13), we focused on the principle of actionable knowledge and avoided theoretical restrictions by using an iterative and pragmatic approach for data analysis (81). Rather theoretical underpinnings were used in an exploratory manner to interpret our findings. We integrated the three legacies of category-centred, case-centred and narrative qualitative methods by performing deductive as well as deductive reasoning (82, 83). This data analysis was based on useful knowledge as posed by the respondents, enabled us to connect pre-existing values, behaviour and identity-based constructs into one framework. While evaluating the MEeP framework (15), qualitative methods enabled deductive and inductive explorations of complex human phenomena with an emphasis on the theoretical underpinnings of TPB and MEeP framework constructs. Quantitative methods (TPB survey) complemented such explorations by enabling the testing of hypotheses arising from qualitative research.

Mixed-methods research can be framed in the context of multiple paradigms like; pragmatic, transformative, post-positivist, and constructivist (1). However, our choice of pragmatic paradigm helped us unpack the dynamic, iterative analytical process by bringing the interconnectedness between experience, knowing, and acting throughout the analysis and write-up phases. Interconnectedness was prominent by keeping the Delphi respondents in the loop and adhering to a prompt timeline of analysis and timely feedback of the findings in various rounds. Keeping the flexibility and adaptability in focus and using an iterative inquiry process allowed the fluidity of abductive, inductive, and deductive reasoning supporting the emergent ideas and data. The same principle was applied to other phased studies in early publications (12–15). This principle had a significant bearing on our dissemination strategy and the utilization of the research findings. Using the pragmatism inherent focus on practice, we probed new ways of knowing and understanding which showed multiple reverberations for the MEeP framework relevance and utility of our research findings. Using this newly developed and validated MEeP framework, we organized a podcast like panel discussion on e-professionalism where the generational perspective (medical student vs. clinical staff) and utility of the MEeP framework was discussed (Panel discussion can be provided on a reasonable request).

### Rigor in mixed-methods research

As in any research paradigm, the goal is to enhance rigor by reducing the researcher’s bias and improving trustworthiness using a transparent approach. Rigor in research pertains to open critique, explicit, open accessed, and free of bias conclusions drawn from an explicitly stated, transparent and replicable research design (84). Rigor is best achieved fulfilling six criteria starting with a clear purpose, adequate preparation, appropriate methods, significant results with an effective presentation and reflective critique (85). Thoughtful and deliberated planning helps the researcher to envision the goal by clarifying the research question and identifying the concepts. While methodological rigor refers to the systematic manner of data collection and analysis while theoretical rigor is the evaluation of theoretical underpinnings leading to relevance (86, 87). These steps introduce explicitness within the research process. Lincoln and Guba outlined four criteria for the trustworthiness of research; transferability (detailed contextual information to ascertain the applications results to one’s situation), credibility (actual representation of results with supporting evidence), dependability (detailed study process for replication) and conformability (communication to the wider audience without researchers bias) were key parameters used to evaluate qualitative work while validity and reliability were used to assess the quality of quantitative research (88).

## Conclusion

To summarize, the pragmatic research design was a major strength of the MEeP framework journey. Using a well-defined methodology and conceptual framework to shape the study design, data collection and analysis, we applied triangulation from multiple sources including digital natives and immigrants, surveys, published literatures, experts’ opinions, and an educational intervention with a pre-post survey. However, the researchers’ stance may have contributed to social desirability bias due to the sensitive nature of the topics, even though we emphasized the independence of interpretation and the anonymity of data. Finally, publication of this phased research in the form of scholarly published articles in leading and cited international medical education journals speaks volumes towards the pragmatic nature, uniqueness, and novelty of the MEeP framework.

## Data availability statement

The original contributions presented in the study are included in the article/supplementary material, further inquiries can be directed to the corresponding author.

## Author contributions

SSG conceptualized this study idea. She searched, screened, and synthesized the literature, and drafted and revised the manuscript. SSG, DH, MY, and SYG together refined the idea and agreed on the layout and structure of this synthesis. All authors contributed to the article and approved the submitted version.
